# Mindfulness-Based Intervention for People With Dementia and Their Partners: Results of a Mixed-Methods Study

**DOI:** 10.3389/fnagi.2019.00092

**Published:** 2019-04-24

**Authors:** Lotte Berk, Franca Warmenhoven, Annemarie P. M. Stiekema, Kim van Oorsouw, Jim van Os, Marjolein de Vugt, Martin van Boxtel

**Affiliations:** ^1^Department of Psychiatry and Neuropsychology, Alzheimer Center Limburg, School for Mental Health and Neuroscience, Maastricht University Medical Centre, Maastricht, Netherlands; ^2^Department of Educational Development and Research, Faculty of Health, Medicine and Life Sciences, Maastricht University, Maastricht, Netherlands; ^3^Limburg Brain Injury Center, Maastricht University, Maastricht, Netherlands; ^4^Department of Clinical Psychological Science, Faculty of Psychology and Neuroscience, Maastricht University, Maastricht, Netherlands; ^5^Department of Psychiatry, Brain Center Rudolf Magnus, University Medical Centre Utrecht, Utrecht, Netherlands; ^6^Department of Psychosis Studies, Institute of Psychiatry, King’s Health Partners, King’s College London, London, United Kingdom

**Keywords:** mindfulness, dementia, caregivers, mixed-methods, MBSR

## Abstract

**Objective:**

Studies have shown preliminary support for mindfulness-based interventions benefitting people with dementia and their caregivers. However, most studies focus on these two groups separately. This study examined whether it would be possible and beneficial for people with dementia and their caregiver to jointly undergo an adjusted Mindfulness-Based Stress Reduction (MBSR) training, named TANDEM.

**Methods:**

The 8-week MBSR training was adjusted based on a literature review and interviews with experts (clinicians and mindfulness trainers). Seven couples (a person with early-stage dementia and their caregiver) participated together in the 8-week TANDEM program. Semi-structured qualitative interviews were conducted after completion. Questionnaires (administered before and after the intervention) assessed the primary outcomes of quality of life and psychological distress (stress, anxiety and depressive symptoms). Secondary outcomes were mindfulness, self-compassion, positive mental health, worrying, and perceived burden (for caregivers).

**Results:**

All participants completed the program and reported beneficial effects (relaxation, awareness, acceptance, and resilience). Most managed to integrate exercises into their daily lives and planned to continue their practice. Participating in a group was considered valuable and supportive. Furthermore, it was appreciated that participants could follow the training together (as a couple). The quantitative results showed a small effect on increased quality of life for caregivers. No substantial decrease in psychological distress was apparent. Caregivers displayed a large increase in mindfulness.

**Conclusion:**

The results of this mixed-methods study suggest that an adjusted mindfulness program is feasible and well-received among couples of persons with early-stage dementia and their caregiver, warranting further research in this area.

## Introduction

Dementia is a worldwide public health priority that affects 35.6 million people worldwide and this number will double in 2030 (World Health Organization [WHO], 2012). Receiving a diagnosis of dementia has a major impact on psychological well-being, both for the persons with dementia as well as the informal caregivers (Bamford et al., 2004). Informal caregivers, often the partner or a relative, fulfill an important role in the care for a person with dementia (PwD). Caring for a PwD is associated with higher levels of distress, illness and a decreased quality of life (Schulz and Martire, 2004). Caregivers have a high risk of developing a depressive disorder (Cuijpers, 2005). It is crucial for both caregivers and PwDs to learn to adapt to the changes the diagnosis of dementia brings and learn to cope with the physical, emotional and social challenges they face.

Interventions to support caregivers and PwDs often focused on each category separately, rather than including a dyad (a PwD and their caregiver together) (Schulz et al., 2007). However, a jointly experienced intervention to support dyadic well-being may be more effective. Dyadic approaches in interventions for chronic disease are more effective than those only focused on patients (Monin and Schulz, 2009). Mindfulness-based interventions represent a promising option to support PwDs and caregivers together. Mindfulness is a skill that can be developed with a training and is defined as an awareness that arises through paying attention, on purpose, in the present moment, and non-judgmentally (Kabat-Zinn, 1990). Mindfulness-based interventions have shown to improve psychological well-being in both healthy and clinical populations (Fjorback et al., 2011; Hempel et al., 2014; Khoury et al., 2015). Most common variations are the Mindfulness-Based Stress Reduction (MBSR; Kabat-Zinn, 1990) and Mindfulness-Based Cognitive Therapy (MBCT; Segal et al., 2002). Both are an 8-week group training where participants practice mindfulness meditation. Mindfulness-based interventions fit in the recent shift to define health from a positive perspective; as an ability to adapt and self-manage in the face of social, physical, and emotional challenges (Huber et al., 2011). This is particularly relevant to a PwD and caregiver facing a future with no cure possible.

Because of the growing evidence of the positive impact of the MBSR and the MBCT on well-being, recent studies have investigated the effects on caregivers, as well as people with mild cognitive impairment and dementia. Although this research is still in its infancy, studies suggest that mindfulness-based interventions could benefit both PwDs and caregivers (Berk et al., 2018). Support for this comes from studies of mindfulness-based intervention with caregivers of PwDs. For example, Moreover, studies that include caregiver and patient dyads have been successful. For example, a mixed-methods study with an MBSR for cancer patients and their partners showed that caregiver burden decreased among partners (van den Hurk et al., 2015). Moreover, both patients and partners indicated in the interviews that they gained more insight into their thoughts, feelings and bodily sensations. Importantly, they reported that it was helpful to participate together with their partner, and that the training led to better mutual understanding. Moreover, a randomized controlled a pilot randomized controlled trial of MBSR for caregivers of family members with dementia showed lower levels of perceived stress and mood disturbance after the training compared to a social support control group (Brown et al., 2016). A feasibility pilot study showed that a mindfulness intervention for PwDs in care homes showed an increase in quality of life (Churcher Clarke et al., 2017).

While these studies have provided tentative support for the effectiveness in both groups separately, more research is needed to investigate if it is feasible to have the caregiver and the PwD participate together in a group training. Moreover, qualitative research can explore the experience of PwDs and their caregivers and the mechanisms through which mindfulness may bring about improvement. The current pilot study used a mixed-method approach to investigate whether (1) an adjusted MBSR program is a feasible intervention for PwDs and their caregivers and (2) whether this MBSR program improves the quality of life and reduces psychological distress in PwDs and their caregivers. We expected that the MBSR program would be feasible and acceptable for PwDs and their caregivers. We expected that participants would report an overall positive experience and examples of how the training helped them to deal with difficult situations. Furthermore, we expected that questionnaires would show increased scores in quality of life and reduced scores on psychological distress.

## Materials and Methods

### Study Design

To explore the feasibility and effectiveness of and adapted MBSR program in PwD and caregivers, a mixed-methods pilot study was conducted. Participants completed self-report questionnaires within 2 weeks before and after the TANDEM program. The questionnaires were complete online, or when requested (*n* = 4) on paper. The semi-structured interviews were conducted individually, 1–3 weeks after the intervention. The interviews took place at the participants’ homes, or when requested (*n* = 2) in a quiet room at the University. The list of topics (see Table 1) was used as a guideline for the interview. The interviewer made sure all topics were discussed, but there was room for the order of topics to be changed if the participant started discussing one of the topics spontaneously. The Ethical Review Committee Psychology and Neuroscience of Maastricht University approved the protocol. All participants gave written informed consent.

**Table 1:** Topic list of semi-structured interview.

(1) General impression
(2) Beneficial and difficult aspects of the training
(3) Materials (i.e., workbook), difficulty
(4) Length of the sessions and training
(5) Home practice
(6) Changes due to the training (i.e., dealing with difficulties)
(7) Future (i.e., plans to continue practice)
(8) Participation with peer group
(9) Participation with partner
(10) Recommendations for adaptations

### Participants

Participants were recruited through regional case managers, flyers, and advertisement in a local paper. Participants were included when they were part of a dyad consisting of a person diagnosed with any form of dementia, and a caregiver. The diagnosis of dementia was determined by self-report indicating that they had received the diagnosis from a medical specialist. Other inclusion criteria included (1) the ability of the PwD to participate in the training, assessed during an intake with the trainer which included a short mindfulness exercise (2) able to understand and use the Dutch language (3) no psychiatric comorbidity requiring medical treatment (4) willingness to attend the training together (5) participation in at least 6 out of 9 sessions. Both trainings took place in the homelike environment of regional centers for informal care for people with cancer.

### Adapted MBSR Program: TANDEM

The TANDEM training (an acronym that, in Dutch, stands for “Attention Training for People with Dementia and their Caregivers”) was based on the original MBSR program as developed by Kabat-Zinn (1990). Several adjustments were made to make the intervention more suitable for PwD and caregivers. The workbook materials were written in an easy to follow language and icons were used to mark different sections. Theme cards were developed to accompany the workbook. Also, topics such as acceptance and communication were given extra time in the training since this was deemed an important theme for PwD and caregivers. Moreover, the movement exercises were modified to be less strenuous. Moreover, the exercise breathing space, a short meditation, from the MBCT was added (Segal et al., 2002). Table 2 shows the content of the TANDEM program per session. The program was discussed with mindfulness trainers and experts on dementia and caregivers. The TANDEM program consisted of eight weekly sessions of 2.5 h each, a 4-h silent day and daily homework assignments of 45 min per day. A range of exercises were taught during the program to cultivate mindfulness, such as the body scan, sitting meditation, gentle yoga exercises, mindful walking, and the 3-min breathing space. During the meetings, home practice and the application of mindfulness in daily life were discussed as well as psycho-education about stress and communication.

**Table 2:** Content of the TANDEM program per session.

Theme	Meditation exercise	Teachings	Homework
(1) Automatic pilot	• Body scan	• Raisin exercise	• Body scan • Eating with awareness • Routine activity with awareness • How to plan homework
(2) Observing clearly	• Body scan • Movement with awareness (short) • Sitting meditation	• 7 pillars of mindfulness • Visualization exercise to demonstrate connection between thoughts and feelings	• Body scan or sitting meditation • Routine activity with awareness • Fill out daily the Pleasant Events Calendar
(3) Attention in movement	• Yoga • Walking meditation • 3-min breathing space	• Exploring boundaries, dealing with limitations	• Body scan or yoga • Breathing space • Fill out daily Unpleasant Events Calendar
(4) Being present	• Sitting meditation • Partner yoga • 3-min breathing space coping	• Psychoeducation about stress • Interrelatedness of feelings, thoughts and bodily sensations	• Yoga • Breathing space (coping) • Awareness of stress reactions
(5) Allowing and seeing what is present	• Sitting meditation • 3-min breathing space coping	• Psychoeducation about acceptance	• Yoga or sitting meditation • Breathing space (coping) • Awareness of communication difficulties
(6) Dealing with difficulties and mindful communication	• Sitting meditation with a difficult situation • Compassionate touch meditation • 3-min breathing space coping	• Exercise and psychoeducation on thoughts aren’t facts • Mindful communication in general and with partner	• Yoga or sitting meditation • Breathing space (coping) • Awareness of communication
Silent day	• Varying meditation exercises • Silent lunch		
(7) Taking care of yourself	• Sitting meditation • 3-min breathing space	• Exercise on evaluating nourishing and depleting activities to improve balance in life • Stress signals (vicious flower)	• Stress signals form • Reflect on training • Plan for continuing practice • Breathing space with action
(8) The rest of your life	• Body scan	• Reflection on training • How to maintain practice	• Write down mindfulness reminders • Letter to yourself

Participants received a workbook with information for each session and a USB or CD with recordings to guide home practice. The two trainers of the TANDEM program were qualified and experienced mindfulness trainers.

### Measures

#### Feasibility and Acceptability

Feasibility was measured with attendance and completion of the program. Acceptability was assessed with semi-structured interviews, in which participants were asked about their experiences and provided feedback (see Table 1).

#### Quality of Life

Quality of life was the primary outcome measure and was assessed using the World Health Organization Quality of Life assessment (WHOQOL-Bref; The WHOQOL Group, 1998; Trompenaars et al., 2005). The four categories physical health, psychological health, social relationships, and environment were averaged to calculate a total quality of life score.

#### Caregiver Burden

The Self-Perceived Pressure from Informal Care (SPPIC), a nine-item questionnaire, was used to assess the extent to which caregiving was experienced as a burden (Pot et al., 1995). To add positive aspects of caregiving, the subscale Care-Derived Self Esteem of the Caregiver Reaction Assessment (CRA-SE) was added (Given et al., 1992).

#### Self-Compassion

Self-compassion was measured using a short 12-item form of the Self-Compassion questionnaire (Neff, 2003; Raes et al., 2011).

#### Positive Mental Health

The Dutch Mental Health Continuum Short Form (MHC-SF) is a 16-item questionnaire that was used to measure emotional, psychological and social well-being (Keyes, 2002; Lamers et al., 2011).

#### Worry

The 16-item *Penn State Worry Questionnaire (PSWQ)* was used to measured worry (Meyer et al., 1990; van der Heiden et al., 2009).

#### Psychological Distress

The 21-item Depression Anxiety Stress Scales (DASS-21) was used to measure psychological distress (Henry and Crawford, 2005).

#### Mindfulness

The Five Facet Mindfulness Questionnaire-Short Form (FFMQ-SF) was used to assess mindfulness (Bohlmeijer et al., 2011). The FFMQ-SF has been validated in older adults (Brady et al., 2018).

### Statistical and Qualitative Analysis

Data were analyzed using SPSS 22.0 (IBM Corporation, Armonk, NY, United States). Cohen’s *d_z_*, the standardized mean difference effect size for within-subject designs, was calculated (Lakens, 2013). No other formal statistical evaluations were calculated, given the explorative nature of the study, insufficient power and absence of a control group.

The content of the interviews was analyzed using deductive content analysis. The transcripts were coded independently by two researchers (LB and AS), using the qualitative software package ATLAS.ti (Scientific Software Development GmbH, Berlin). The codes where clustered within categories, based on the semi-structured interview, independently and discussed to reach consensus. This consensus process was verified by a third researcher (MdV).

## Results

### Participants

A total of 14 people participated in the training, or seven couples. All caregivers were the partners of the PwD. The age of participants ranged from 56 to 81 years. Table 3 gives an overview of participants’ characteristics.

**Table 3:** Characteristics of people with dementia and partners at baseline.

	Total (*n* = 14)	Persons with dementia (*n* = 7)	Partners (*n* = 7)
Age, mean (*SD*)	71.11 (7.45)	71.46 (7.41)	70.75 (8.14)
Gender (*n*, % female)	7 (50)	2 (29)	5 (71)
Level of education (*n*, %)			
Low	3 (21)	1 (14)	2 (29)
Middle	8 (57)	4 (57)	4 (57)
High	3 (21)	2 (29)	1 (14)
Type of dementia^a^ (*n*, %)			
Alzheimer’s disease		4 (57)	
Vascular dementia		2 (29)	
Frontotemporal dementia		1 (14)	
Time since diagnosis (in years), mean (*SD*)		1.89 (1.69)	

*^a^For some participants the diagnosis was probable AD and they did not pursue further diagnostic procedures.*

### Feasibility

All participants attended six or more sessions of the TANDEM training, with an average of 7.3 out of the eight weekly sessions. All participants completed the post-training questionnaires.

Five out of seven participants with dementia indicated that their partner had helped them with filling out the questionnaires.

### Qualitative Evaluation

All participants completed the interviews after the training ended, with the exception of one couple. They had received bad news and did not want to participate in the interviews anymore but did fill out the questionnaires. Their case-manager reported that they had enjoyed the training, in particular that all participants were respected, the materials were good, and that many relevant topics were discussed. A total of twelve participants were interviewed.

The results from the interviews were divided into four topics: experiences, participating with partner, effects, and practical feasibility. These topics are described below and clarified by quotations from the interviews (C, caregiver; PwD, person with dementia).

#### Experiences

In general, participants were very positive about the TANDEM training. Participants mentioned several aspects they appreciated about the training such as the good atmosphere in the group, pleasant location, practical, logical structure, sharing experiences with group members, becoming calmer, and the training being very useful in general. All participants expressed satisfaction with their trainer. They felt supported and connected because of the trainer, and the trainer’s voice was experienced as pleasant.

“I was really impressed [with the training]. In particular, the complete structure of the training, the direct applicability of everything, the space that everyone had to express themselves. I have the feeling that all those other couples, that we really got to know them… There is truly a back-and-forth. The homework. Everything felt right.” (C3)

“[The trainer was great because] she was very harmonious and friendly in the way she interacted with us. …. But what we also experienced (when we were at home, put the iPad on the table, and sat down to do the exercises) that she had such a pleasant voice. Yes, that did a lot for me.” (PwD3)

Participants started the training with different expectations. Some participants had the need to learn new tools to cope with the diagnosis of dementia, or to get help with specific problems (e.g., difficulty sleeping). Other participants had some doubts and started the training with a critical attitude because they did not know if and how it could help them or thought it might be too ‘new-age’ for them.

“I actually started [the training] reluctantly. We had signed up for it. …. Thinking, ‘We will at least start it. Start it, try it. And then see if it does anything for us.”’ (C6)

Different experiences with practice during the training were reported. Whereas a few participants found a daily routine to practice, most did not practice every day and tended to prefer shorter exercises such as the 3-min breathing space. Participants experienced relaxation, falling asleep during practice, and having difficulties doing the exercises at home. The longer exercises (45 min) were more difficult to plan but were recognized as beneficial.

“The longer exercises had a high threshold, so I had a hard time getting started. Even though I would be the first one to say, ‘If you start such a long exercise and you experience the calmness that comes with it, it is very pleasant.’ Even though the cost was high, the reward was definitely there.” (C4)

One participant frequently experienced sadness while practicing. Although she appreciated a new-found connection with her emotions, she also struggled with how to deal with this.

“And then you got the assignment to see if you could apply the exercises when things don’t go well. ‘What do you feel? How does that feel? What stands in the way? And if something is in the way, do nothing with it. It is there and it can be there.’ And what happened with me, in that phase of the training, is that I was confronted with my sadness. …. As a rational human being you try to solve things that are unsolvable. You can be confronted with things that you can solve. But not the whole story [of dementia]. And at first you try to reflect on it as little as possible. But that is doomed to fail, because that isn’t how it works. You try to find a way … you can’t get around it. So, I increasingly got very sad, but not in a negative way.” (C4)

#### Participating in a Group With Their Partner

All participants felt positive about participating in a group, which was a safe place where one could be oneself and share one’s experiences. The group was considered a very valuable aspect of the training; participants felt connected to the group members and felt less alone because of them. Some participants shared more in the group than others. A few participants noticed differences when comparing themselves to the group. One PwD noticed that other PwDs were worse off than he was. One caregiver noticed that everyone was dealing with the diagnosis in their own way.

“[Participating in a group] was marvelous… Of course, it is in the back of your mind, ‘what will happen [in the future regarding the diagnosis]?’ … The fear. And then you hear from other people how they felt and what they experienced. I learned a lot and I got a lot out of it.” (C1)

“We were a good group together. It felt right for us. We got along. We felt a camaraderie. It really was good. Everyone shared with each other except for me. I’m not much of a talker.” (PwD1)

Participation of both PwD and partners in one group was perceived as valuable. Couples enjoyed it. Some mentioned a feeling of solidarity and liking that their partners heard their experiences in the group. None of the participants felt the need to do the training without their partner. One caregiver mentioned that even though she could imagine that a group with only caregivers could be valuable, she would still prefer doing the training with her partner. One PwD was concerned before the training started that his partner would say negative things about him during the training.

“Well, what went through my mind [before the training started] was, ‘She’s not going to say unpleasant things about me, is she?’ That wasn’t the case.” (PwD3)

“[Participating with my partner was] very pleasant. Just because we can talk about it well, doesn’t mean that everything comes up in conversation. Just because it doesn’t come up. Not because it’s taboo. But a lot of things come up [in the training] that made me think, ‘that’s good for him to hear.”’

Couples practiced together as well as separately. They encouraged each other to do the exercises. One caregiver had a hard time not getting angry when her partner did not want to do the exercises with her.

Most of the time he reminded me [to do the exercises]. (laughter) I would think, ‘Should I skip today.?’ …. But he was the instigator. (PwD6)

We did the [homework] together. After the morning coffee, my husband was the one to get everything ready, as if to say, ‘come on.’ I really liked that I didn’t have to make him do it.” (C3)

Some participants reported that the training had influenced their relationship as a couple. Caregivers were better able to prevent or deal with quarrels. They felt more connected. One caregiver mentioned that they had more physical contact. Several participants mentioned that the communication between them had improved.

“[My partner] is very fast. When she talks she jumps from one thing to the next. [I’ll say:] ‘What are you talking about?’ ‘Oh, wait,’ she says. She didn’t used to do that. That has completely changed over the last month. …. She says, ‘Let me explain,’ and then she does explain it. And she really does. And that makes her so much happier, and me too. So that works. Not always. But usually we do it like that.” (PwD4)

“I ask him more questions. I don’t assume as much anymore. And I used to be very good at assuming. And now I just ask, ‘What is that like for you?’ This is much more useful to him. He participates more. That has changed.” (C3)

#### Effects

The training brought beneficial changes to all participants, with the exception of one PwD who said that nothing had changed. Participants mostly report increased calmness and relaxation. The training helped both caregivers and PwD to cope with and accept the diagnosis. They reported an increased awareness and spending less time on automatic pilot and instead being in the moment. Some participants reported increased self-care. Moreover, participants learned new ways to deal with difficulties.

“At night, sometimes I wake up in a panic, and I used to stay up all night… awake with palpitations and feeling miserable. But now I know I can’t change it. There’s nothing that I can do about the situation. It is how it is and it will follow its course. And then I just focus on my breath. And I know I can’t do anything to change this. This doesn’t make it painless, but it helps me to keep going.” (C3)

“I notice that I react better to the outside world. I’m on the board of a community center. Someone else on the board [said about me,] ‘Typical, he probably forgot.’ That gets under my skin…. It makes me mad. But now, mindfulness makes me calmer.” (PwD2)

“I feel better. I’m a nervous Nellie. I’m someone who likes to be on time. I can’t stand being late. That makes me a little nervous, but it got better. …. I think, ‘Oh, let it go.’ (PwD5)

#### Practical Feasibility

The materials (the workbook and cards) were appreciated, however, most PwD did not use them. Some caregivers used the workbook but most considered it a reference book and did not use it much. The workbook was considered legible and accessible. Moreover, a few caregivers mentioned that the texts and poems were inspiring. The cards were put up in their houses as a reminder by most participants.

“[Reading] was difficult. I would read [the cards], but then I would forget what it said. They weren’t really useful.” (PwD4)

“We always put the cards on the table, every week a different one. It reminds you…a few sayings stick with you: [Grant me the serenity to accept the things I cannot change, courage to change the things I can, and wisdom to know the difference.]” (C2)

The length of the 8-week training was considered adequate by most participants. One participant would not mind if it were shorter, whereas another expressed a strong need for continued support with the practice. Although most participants would enjoy booster sessions the general impression was that the length was appropriate.

“The 8 weeks. That was not something I was looking forward to, but apparently it was necessary to get through the material. In hindsight, I liked going. Last week [I was disappointed that it had ended]. Now I’m happy to have time for other things.” (C5)

“Before [the training started], I thought to myself, ‘Geez Louise, busy for 2 months!’ And then you’re also supposed to [do homework.] It wasn’t as if I didn’t want to do it, but I wasn’t exactly enthusiastic. But already after the first meeting I wanted to continue. Yes, it was long, but I liked it very much.” (PwD3)

The duration of the sessions (2.5 h) was considered appropriate and feasible.

“I thought that [the length of the session] was good. At a certain moment it’s enough. Your head is full. Full or empty. You start to notice one thing or another, and then I think it’s enough. Then you want to go and digest it.” (C2)

Different elements from the training were considered most useful, but only after the initial response that everything was considered useful. Participants mentioned elements such as taking care of oneself and finding a balance between activities that consume or give energy, calmness that the exercises bring, the 3-min breathing space because it is easily applicable, the explanation about acceptance, and remembering quotes like “You can’t stop the waves, but you can learn to surf” when dealing with difficult situations. One caregiver had an immediate answer when asked what was most useful, namely the analogy of biking on a tandem together.

“[The analogy of riding a tandem bicycle together] got through to me the most of anything in the training. I thought, ‘Dang, that’s it!’ … This is what’s happening. You are on the back seat of a tandem bicycle. You have to follow. Unfortunately, you have to follow, because she will not get better, it will only get worse. So, you will have to give in. You will have to accept it. That is difficult, but because of the training I have arrived at that insight.” (C6)

Participants had a difficult time mentioning what specifically from the training was least useful. They responded that nothing in particular could be omitted. After that, sometimes a few personal experiences were mentioned such as disliking doing exercises on the floor or already having the knowledge of certain topics discussed. However, nobody reported that anything should be left out of the program.

“I worked in education and have experience with communication. That part [of the training] wasn’t an eyeopener for me. …. Then you know how communication is heavily influenced by assumptions…. But it is good to have it in [the training], because there will be a lot of people that do not realize this.” (C4)

When asked specifically if they thought adjustments were necessary, most participants did not have a specific answer. A few people wished that the training was closer to where they lived, that it would be helpful if there would be booster sessions, that the group should probably not exceed 10 people, and that health insurance should cover this training for caregivers and PwD. All participants had the intention to keep up with their practice, although one PwD would let it depend on his partner. In particular the breathing space and informal exercises (e.g., practicing awareness in daily activities) were mentioned.

### Effectiveness

Table 4 shows the scores of the questionnaires before and after the TANDEM training. Caregivers’ and PwD scores show different directions in changes. Changes with a large effect size (*d_z_* ≥ 0.8) were found with increased mindfulness in caregivers and reduced self-compassion in PwD. Small (*d_z_* = 0.2) to medium (*d_z_* = 0.5) effect sizes were found for reductions in mental health and quality of life in PwD, and reductions in worry and self-esteem, and an increase in quality of life in caregivers.

**Table 4:** Characteristics, baseline and post-intervention scores of persons with dementia and partners.

	Total					Persons with dementia					Partners				
	Baseline (*n* = 14)		Post (*n* = 14)			Baseline (*n* = 14)		Post (*n* = 14)			Baseline (*n* = 14)		Post (*n* = 14)		
	*M*	*SD*	*M*	*SD*	*d_z_*	*M*	*SD*	*M*	*SD*	*d_z_*	*M*	*SD*	*M*	*SD*	*d_z_*
QoL (WHOQOL-Bref)	16.01	1.34	16.07	1.33	0.09	16.43	1.33	16.26	1.74	0.29	15.51	1.31	15.85	0.70	0.47
Psychological distress (DASS-21)	8.48	7.20	8.14	8.03	0.03	6.00	5.05	6.29	5.77	0.04	10.95	8.51	10.00	9.91	0.07
Mindfulness (FFMQ-SF)	81.92	6.74	83.85	7.10	0.39	83.00	6.32	82.17	7.25	0.13	81.00	7.44	85.29	7.20	2.86
Self-compassion (SCS-SF)	3.49	0.84	3.29	0.45	0.23	4.01	0.63	3.22	0.51	1.11	3.05	0.76	3.35	0.43	0.39
Mental health (MHC-SF)	3.64	1.04	3.60	0.61	0.07	3.85	0.47	3.63	0.40	0.52	3.44	1.42	3.57	0.81	0.17
Worry (PSWQ)	48.71	11.18	48.43	12.90	0.03	42.43	11.76	44.57	11.72	0.18	55.00	6.35	52.29	13.73	0.25
Caregiver burden (SPPIC)											26.14	7.73	26.71	7.18	0.17
Caregiver self-esteem (CRA-SE)											24.57	4.86	23.57	4.58	0.46

*d_z_, Cohen’s d standardized mean difference effect size for within-subject designs; CRA-SE, Caregiver Reaction Assessment - Care-Derived Self Esteem subscale; DASS-21, 21-item Depression Anxiety Stress Scale; FFMQ-SF, Five Facet Mindfulness Questionnaire - Short Form; PSWQ, Penn State Worry Questionnaire; MHC-SF, Mental Health Continuum - Short Form; QoL, Quality of Life; SCS-SF, Self Compassion Scale - Short Form; SPPIC, Self-Perceived Pressure from Informal Care; WHOQOL-Bref, World Health Organization Quality of Life assessment.*

## Discussion

This study showed that participating in the TANDEM training, an adjusted MBSR, was feasible for PwD and their partners. All participants completed the training. Quantitative data showed a small effect for an increase in quality of life in caregivers and a decrease for PwD. Participants did not show a substantial reduction in psychological distress, but the levels were not elevated at baseline. Caregivers showed a large increase in mindfulness. No large effects were found for PwD. The qualitative analysis showed that for most participants, the training seemed to increase calmness, awareness, acceptance, and resilience (e.g., dealing with difficult situations). Participating in a group together with their partner was considered very valuable and had a positive influence on their relationship. These results suggest that the training provides tools to both PwD and caregiver to cope with dementia and to support each other.

In a qualitative study on the needs of early-stage dementia caregivers, caregivers reported the importance of acceptance to be able to adapt to the situation (Boots et al., 2015). However, caregivers also indicated the lack of knowledge, difficulty acknowledging changes, and focus on loss interfered with the process of acceptance. In our study, participants reported that acceptance was what they gained from the TANDEM training. It seems that the training might fulfill an important aspect needed to adapt to the disease.

Increased acceptance can potentially influence caregiver management strategies. Caregiver management strategies can be characterized by a high or low level of acceptance of the caregiving situation and dementia related problems (de Vugt et al., 2004). Future research could investigate whether the TANDEM training might positively influence a non-adaptive care management strategy that is characterized by a low level of acceptance and high level of neuroticism in the caregiver. Mindfulness has an inverse association with neuroticism (Giluk, 2009). Successful interventions that target neuroticism and a non-adaptive caregiver strategy could result in lower levels of negative emotions, such as depression, in the caregiver and lower levels of behavioral changes, such as hyperactivity, in the person with dementia (de Vugt et al., 2004).

All participants felt positive about the trainer. Even though the groups had two different teachers, similar aspects were mentioned such as liking the voice and the way the trainer interacted with the group. It is likely that the trainer is a crucial element in the training, by facilitating the group process and providing a safe environment. Although elaborate teacher training and meditation experience is considered essential to ensure that the mindfulness training is delivered properly and effectively (Crane et al., 2017), little research has been done on the importance of trainer experience and competence. Recently, one study showed that trainer competence of MBCT was not associated with treatment outcome for depression (Huijbers et al., 2017), whereas another study showed that higher training levels of teachers were associated with positive outcomes in MBSR (Ruijgrok-Lupton et al., 2018). Although more research is needed, we recommend a trainer with in-depth teacher training and experience.

None of the participants mentioned that they would rather participate without their partner. However, it might be possible that caregivers would benefit from participating in a group with other caregivers only, since it is possible that during the training they are more preoccupied with the well-being of their partner instead of their own. A pilot study with lung cancer patients and caregivers showed that participants can feel distracted by the presence of their partner because they are concerned about their well-being (van den Hurk et al., 2015). The participants in our study did not report this, however, future studies should keep this potential issue in mind. Moreover, future research could investigate how the connection between PwD and caregiver will change over time while the illness progresses. Mindfulness may offer a tool to increase resilience to the unavoidable changes in their relationship that come with a diagnosis of dementia. It may offer people the flexibility to be with the loss of the known relationship and a willingness to explore love and connection in the changed relationship. Furthermore, future research could investigate the influence between the outcome measures in the dyads. That is, is it more likely that both the PwD and caregiver show improvements on the measurements, or do discrepancies (e.g., one of them shows a large change but not the other) exist?

One participant reported that the training made her experience sadness. This was not a negative experience, but she did not know if it was going to pass. Although this is the experience of a single individual, it should be noted that the training might increase negative emotions by providing mental space for these during practice. It is crucial that trainers are aware of this possibility and that the participants are guided in this process.

The study has several limitations. The results may reflect a self-selection bias; where certain characteristics may motivate people to participate in the mindfulness training. Future research could interview potential participants that decide not to participate to get more insight into barriers to participate in a mindfulness training. Recruitment was not particularly easy for this study because both the PwD and caregiver had to be willing to participate. Moreover, in general it seemed that for many potential participants the time commitment prevented participation. Many PwD (71%) indicated that their partner helped them filling out their questionnaires. Therefore, it is unclear whether this might have influenced their answers to the questions. This study was a feasibility pilot without a control group. However, previous research has compared an MBSR training with an active control. A pilot study with an adapted MBSR for caregivers of PwDs showed a decrease on caregiver stress also for the active control compared to the respite-only group (Oken et al., 2010). Future research could consider an active control group such as the Health Enhancement Program, which is similar to the MBSR but without the mindfulness component (MacCoon et al., 2012). Our study did not investigate the amount of practice time and perceived benefit. Future research could measure adherence to suggested homework and the relationship with outcome.

One of the strengths of the study is its mixed-methods design. By using both qualitative and quantitative methods we get better insight into whether this training is feasible and effective. Monitoring attendance and completion rates is indicative of the acceptance of the training, while information from the interviews can give more in-depth information on why participants completed the training and what might need to be adjusted in the future. This study showed that it is possible to having PwDs and their partners participate in a group together. This is in line with previous research showing feasibility with patients with progressive cognitive decline and their caregivers (Paller et al., 2015). Although our study had a small sample size, reporting quantitative data can help future larger studies with power calculations. Moreover, comparing the results from the questionnaires and interviews, we can see whether they overlap and what might not be registered by questionnaires. The quantitative results did not show large changes in psychological distress or worry, even though participants indicate in the interviews many positive effects such as increased calmness, acceptance, and ability to deal with difficult situations. Mindfulness exercises have given them a tool whenever things get too much. Apparently, these benefits are difficult to assess with the outcome measures that were chosen. Our study showed a small increase in caregiver burden, and a small decrease in caregiver self-esteem. These results are in the opposite direction of what was expected. The standard deviation indicates that there was a high variability. This was not only the case for these two questionnaires, but for many other questionnaires. Participants might differ a lot in the changes they report. Future research might look into other options than retrospective questionnaires, such as experience sampling methodology to get more insight into individual processes (van Os et al., 2017). Moreover, other questionnaires, such as the Applied Mindfulness Process Scale might be useful to get insight into how participants use their mindfulness practice to deal with negative or stressful events in daily lives (Li et al., 2016).

To conclude, this study demonstrates that TANDEM, an adapted MBSR, is a feasible intervention for people with dementia and their caregivers, together in one group. Quantitative data showed a small effect on increase in quality of life for caregivers and a decrease for PwD, but no effects on psychological distress. Although specific benefits differed between participants, many beneficial aspects (increased relaxation, awareness, acceptance, and resilience) were shared by most caregivers and PwD. The training might help people with dementia and their caregivers to accept the diagnosis and deal with the changes ahead. Future research combining experience sampling methodology and qualitative research is important to obtain further insight into how to tailor interventions for this population.

## Ethics Statement

This study was carried out in accordance with the recommendations of the Ethical Review Committee Psychology and Neuroscience of Maastricht University with written informed consent from all subjects. All subjects gave written informed consent in accordance with the Declaration of Helsinki. The protocol was approved by the Ethical Review Committee Psychology and Neuroscience of Maastricht University.

## Author Contributions

All authors listed have made a substantial, direct and intellectual contribution to the work, and approved it for publication.

## Conflict of Interest Statement

The authors declare that the research was conducted in the absence of any commercial or financial relationships that could be construed as a potential conflict of interest.

## References

[B1] Bamford C. Lamont S. Eccles M. Robinson L. May C. Bond J. (2004). Disclosing a diagnosis of dementia: a systematic review. *Int. J. Geriatr. Psychiatry* 19 151–169. 10.1002/gps.1050 14758581

[B2] Berk L. Warmenhoven F. van Os J. van Boxtel M. (2018). Mindfulness training for people with dementia and their caregivers: rationale, current research, and future directions. *Front. Psychol.* 9:982. 10.3389/fpsyg.2018.00982 29951027 PMC6008507

[B3] Bohlmeijer E. ten Klooster P. M. Fledderus M. Veehof M. Baer R. (2011). Psychometric properties of the five facet mindfulness questionnaire in depressed adults and development of a short form. *Assessment* 18 308–320. 10.1177/1073191111408231 21586480

[B4] Boots L. M. M. Wolfs C. A. G. Verhey F. R. J. Kempen G. I. J. M. de Vugt M. E. (2015). Qualitative study on needs and wishes of early-stage dementia caregivers: the paradox between needing and accepting help. *Int. Psychogeriatr.* 27 927–936. 10.1017/S1041610214002804 25566686

[B5] Brady B. Kneebone I. I. Bailey P. E. (2018). Validation of the five facet mindfulness questionnaire among community-dwelling older adults. *Mindfulness* 10 1–8. 10.1007/s12671-018-0994-0

[B6] Brown K. W. Coogle C. L. Wegelin J. (2016). A pilot randomized controlled trial of mindfulness-based stress reduction for caregivers of family members with dementia. *Aging Ment. Health* 20 1157–1166. 10.1080/13607863.2015.1065790 26211415 PMC5070659

[B7] Churcher Clarke A. Chan J. M. Y. Stott J. Royan L. Spector A. (2017). An adapted mindfulness intervention for people with dementia in care homes: feasibility pilot study. *Int. J. Geriatr. Psychiatry* 32 e123–e131. 10.1002/gps.4669 28170104

[B8] Crane R. S. Brewer J. Feldman C. Kabat-Zinn J. Santorelli S. Williams J. M. G. (2017). What defines mindfulness-based programs? The warp and the weft. *Psychol. Med.* 47 990–999. 10.1017/S0033291716003317 28031068

[B9] Cuijpers P. (2005). Depressive disorders in caregivers of dementia patients: a systematic review. *Aging Ment. Health* 9 325–333. 10.1080/13607860500090078 16019288

[B10] de Vugt M. E. Stevens F. Aalten P. Lousberg R. Jaspers N. Winkens I. (2004). Do caregiver management strategies influence patient behaviour in dementia? *Int. J. Geriatr. Psychiatry* 19 85–92. 10.1002/gps.1044 14716704

[B11] Fjorback L. O. Arendt M. Ornbøl E. Fink P. Walach H. Fjorback M. (2011). Mindfulness-based stress reduction and mindfulness-based cognitive therapy – A systematic review of randomized controlled trials. *Acta Psychiatr. Scand.* 124 102–119. 10.1111/j.1600-0447.2011.01704.x 21534932

[B12] Giluk T. L. (2009). Mindfulness, big five personality, and affect: a meta-analysis. *Pers. Ind. Diff.* 47 805–811. 10.1016/J.PAID.2009.06.026

[B13] Given C. W. Given B. Stommel M. Collins C. King S. Franklin S. (1992). The caregiver reaction assessment (CRA) for caregivers to persons with chronic physical and mental impairments. *Res. Nurs. Health* 15 271–283. 10.1002/nur.4770150406 1386680

[B14] Hempel S. Taylor S. L. Marshall N. J. Miake-Lye I. M. Beroes J. M. Shanman R. (2014). *Evidence Map of Mindfulness.* Washington, DC: Department of Veterans Affairs (US). 25577939

[B15] Henry J. D. Crawford J. R. (2005). The short-form version of the depression anxiety stress scales (DASS-21): construct validity and normative data in a large non-clinical sample. *Br. J. Clin. Psychol. Soc.* 44 227–239. 10.1348/014466505X29657 16004657

[B16] Huber M. Knottnerus J. A. Green L. van der Horst H. Jadad A. R. Kromhout D. (2011). How should we define health? *BMJ (Clin. Res. Ed.)* 343:d4163. 10.1136/BMJ.D4163 21791490

[B17] Huijbers M. J. Crane R. S. Kuyken W. Heijke L. van den Hout I. Donders A. R. T. (2017). Teacher competence in mindfulness-based cognitive therapy for depression and its relation to treatment outcome. *Mindfulness* 8 960–972. 10.1007/s12671-016-0672-z 28757901 PMC5506231

[B18] Kabat-Zinn J. (1990). *Full Catastrophe Living: Using the Wisdom of Your Body and Mind to Face Stress, Pain, and Illness.* New York, NY: Delacorte.

[B19] Keyes C. L. M. (2002). The mental health continuum: from languishing to flourishing in life. *J. Health Soc. Behav.* 43 207–222. 10.2307/3090197 12096700

[B20] Khoury B. Sharma M. Rush S. E. Fournier C. (2015). Mindfulness-based stress reduction for healthy individuals: a meta-analysis. *J. Psychosom. Res.* 78 519–528. 10.1016/j.jpsychores.2015.03.009 25818837

[B21] Lakens D. (2013). Calculating and reporting effect sizes to facilitate cumulative science: a practical primer for t-tests and ANOVAs. *Front. Psychol.* 4:863. 10.3389/fpsyg.2013.00863 24324449 PMC3840331

[B22] Lamers S. M. A. Westerhof G. J. Bohlmeijer E. T. ten Klooster P. M. Keyes C. L. M. (2011). Evaluating the psychometric properties of the mental health continuum-short form (MHC-SF). *J. Clin. Psychol.* 67 99–110. 10.1002/jclp.20741 20973032

[B23] Li M. J. Black D. S. Garland E. L. (2016). The applied mindfulness process scale (AMPS): a process measure for evaluating mindfulness-based interventions. *Pers. Ind. Diff.* 93 6–15. 10.1016/j.paid.2015.10.027 26858469 PMC4742344

[B24] MacCoon D. G. Imel Z. E. Rosenkranz M. A. Sheftel J. G. Weng H. Y. Sullivan J. C. (2012). The validation of an active control intervention for mindfulness based stress reduction (MBSR). *Behav. Res. Ther.* 50 3–12. 10.1016/j.brat.2011.10.011 22137364 PMC3257026

[B25] Meyer T. J. Miller M. L. Metzger R. L. Borkovec T. D. (1990). Development and validation of the penn state worry questionnaire. *Behav. Res. Ther.* 28 487–495. 10.1016/0005-7967(90)90135-6 2076086

[B26] Monin J. K. Schulz R. (2009). Interpersonal effects of suffering in older adult caregiving relationships. *Psychol. Aging* 24 681–695. 10.1037/a0016355 19739924 PMC2765123

[B27] Neff K. D. (2003). Development and validation of a scale to measure self-compassion. *Self Identity* 2 223–250. 10.1080/15298860309027 26979311

[B28] Oken B. S. Fonareva I. Haas M. Wahbeh H. Lane J. B. Zajdel D. (2010). Pilot controlled trial of mindfulness meditation and education for dementia caregivers. *J. Alternat. Complement. Med.* 16 1031–1038. 10.1089/acm.2009.0733 20929380 PMC3110802

[B29] Paller K. A. Creery J. D. Florczak S. M. Weintraub S. Mesulam M. M. Reber P. J. (2015). Benefits of mindfulness training for patients with progressive cognitive decline and their caregivers. *Am. J. Alzheimer’s Dis. Other Dementias* 30 257–267. 10.1177/1533317514545377 25154985 PMC4363074

[B30] Pot A. M. van Dyck R. Deeg D. J. (1995). Perceived stress caused by informal caregiving. Construction of a scale. *Tijdschr. Voor Gerontologie Geriatrie* 26 214–219. 8750982

[B31] Raes F. Pommier E. Neff K. D. Van Gucht D. (2011). Construction and factorial validation of a short form of the self-compassion scale. *Clin. Psychol. Psychother.* 18 250–255. 10.1002/cpp.702 21584907

[B32] Ruijgrok-Lupton P. E. Crane R. S. Dorjee D. (2018). Impact of mindfulness-based teacher training on MBSR participant well-being outcomes and course satisfaction. *Mindfulness* 9 117–128. 10.1007/s12671-017-0750-x 29387265 PMC5770494

[B33] Schulz R. Hebert R. S. Dew M. A. Brown S. L. Scheier M. F. Beach S. R. (2007). Patient suffering and caregiver compassion: new opportunities for research, practice, and policy. *Gerontologist* 47 4–13. 10.1093/geront/47.1.4 17327535

[B34] Schulz R. Martire L. M. (2004). Family caregiving of persons with dementia: prevalence, health effects, and support strategies. *Am. J. Geriatr. Psychiatry* 12 240–249. 10.1097/00019442-200405000-00002 15126224

[B35] Segal Z. V. Williams J. M. G. Teasdale J. D. (2002). *Mindfulness-Based Cognitive Therapy for Depression: A New Approach to Preventing Relapse.* New York, NY: Guilford.

[B36] Trompenaars F. J. Masthoff E. D. Van Heck G. L. Hodiamont P. P. De Vries J. (2005). Content validity, construct validity, and reliability of the WHOQOL-Bref in a population of Dutch adult psychiatric outpatients. *Qual. Life Res.* 14 151–160. 10.1007/s11136-004-0787-x 15789949

[B37] van den Hurk D. G. M. Schellekens M. P. J. Molema J. Speckens A. E. M. van der Drift M. A. (2015). Mindfulness-based stress reduction for lung cancer patients and their partners: results of a mixed methods pilot study. *Palliat. Med.* 29 652–660. 10.1177/0269216315572720 25701663 PMC4457793

[B38] van der Heiden C. Muris P. Bos A. E. R. van der Molen H. Oostra M. (2009). Normative data for the Dutch version of the penn state worry questionnaire, Netherlands. *J. Psychol.* 65 69–75. 10.1007/BF03080129

[B39] van Os J. Verhagen S. Marsman A. Peeters F. Bak M. Marcelis M. (2017). The experience sampling method as an mHealth tool to support self-monitoring, self-insight, and personalized health care in clinical practice. *Depress. Anxiety* 34 481–493. 10.1002/da.22647 28544391

[B40] The WHOQOL Group. (1998). Development of the world health organization WHOQOL-BREF quality of life assessment. *Psychol. Med.* 28 551–558. 9626712 10.1017/s0033291798006667

[B41] World Health Organization [WHO] (2012). *Dementia: A Public Health Priority.* Geneva: WHO.

